# Psychiatric morbidity among patients tested positive and isolated for COVID-19

**DOI:** 10.1192/j.eurpsy.2021.700

**Published:** 2021-08-13

**Authors:** U. Zubair

**Affiliations:** Oak, phoenix care center, Dublin, Ireland

**Keywords:** COVID-19, psychiatric morbidity, socio-demographic factors

## Abstract

**Introduction:**

In this crisis situation when everybody has been emphasizing on preventive measures, screening, early recognition and provision of necessary equipment, less emphasis has been laid on the mental health of the sufferers who have been listening news and following social media reporting catastrophe linked with COVID 19.

**Objectives:**

To look for the psychiatric morbidity and associated socio-demographic factors among patients tested positive and isolated for covid-19

**Methods:**

All patients tested positive for covid-19 and admitted in Covid-19 ward of Malir hospital without any associated complications were included in the study. General Health Questionare-12 (GHQ-12) was administered to look for the presence of psychiatric morbidity. Chi-square test and binary logistic regression analysis were the tests applied to look for the relationship of various socio-demographic factors with presence of psychiatric morbidity among the target population.

**Results:**

Out of 61 patients included in the study, 45 (73.7%) showed the presence of psychiatric morbidity while 16 (26.3%) did not show psychiatric morbidity when screened with GHQ-12. 43 (70.4%) were male while 18 (29.6%) were female. Mean age of the patients was 35.21 ±2.355 years. Regression analysis revealed that advanced age and female gender have statistically significant relationship (p-value<0.05) with presence of psychiatric morbidity among patients of covid-19.

**Conclusions:**

A considerable number of patients had psychiatric morbidity after being tested positive for covid-19 and isolated in the health facility. Female patients and patients with age more than 40 years were found to be more at risk of developing psychiatric morbidity among the patients admitted in covid-19 ward.

